# Intrauterine hyperglycemia induces intergenerational Dlk1-Gtl2 methylation changes in mouse placenta

**DOI:** 10.18632/oncotarget.23976

**Published:** 2018-01-05

**Authors:** Ying Jiang, Yi-Chen Yu, Guo-Lian Ding, Qian Gao, Feng Chen, Qiong Luo

**Affiliations:** ^1^ Department of Obstetrics, Women's Hospital, School of Medicine, Zhejiang University, Hangzhou, China; ^2^ Department of General Surgery, Institute of Minimally Invasive Surgery, Sir Run Run Shaw Hospital, School of Medicine, Zhejiang University, Hangzhou, China; ^3^ International Peace Maternity and Child Health Hospital, School of Medicine, Shanghai Jiaotong University, Shanghai, China; ^4^ Tianjin Central Hospital of Obstetrics and Gynecology, Reproductive Medical Center, Tianjin, China

**Keywords:** epigenetic regulation, microarray analysis, intrauterine hyperglycemia environment, placenta, intergenerational transmission

## Abstract

An intrauterine hyperglycemic environment has long-lasting effects on the offspring. Recent studies focused on fetal tissues, whereas we studied the development and molecular alteration of the placenta. By intercrossing male and female adult control (C) and first-generation offspring mice with gestational diabetes mellitus (F1-GDM), we obtained four groups of second generation (F2) offspring: 1) C♂-C♀, 2) C♂-GDM♀, 3) GDM♂-C♀, 4) GDM♂- GDM♀. Placental weights in F1-GDM offspring were lower than in the control group. Placental weights in F2-offspring decreased through the paternal line. Placental RNA was extracted and analyzed using microarrays on day18.5 of pregnancy. This revealed 35 upregulated imprinted genes and 10 down-regulated imprinted genes. Dlk1and Gtl2 were especially down-regulated and up-regulated, respectively, due to their abnormal methylation status. These findings suggest that intrauterine hyperglycemia decreased placental weight in the first generation, and this was transmitted paternally to the second generation in mice. They also suggest intrauterine hyperglycemia leads to abnormal placental Dlk1-Gtl2 expression due to DNA methylation in first and second generation mice.

## INTRODUCTION

Gestational diabetes mellitus (GDM) develops in 3% to 10% of pregnant women, and is associated with hyperglycemia during pregnancy [1]. An adverse intrauterine environment will affect multiple generations [2, 3]. Offspring born from a GDM pregnancy suffer long-term outcomes like obesity, hypertension, type 2 diabetes mellitus (T2D) [4–6].

The placenta conducts the maternal-fetal transport of nutrients and gases, and is exposed to the intrauterine conditions which may adversely affect placental and fetal development. This makes it a valuable tissue for investigating the molecular effects of GDM [7, 8]. The placenta is more susceptible to maternal perturbations than the fetus, and complications may be transmitted to the fetus [9–11].

Epigenetic processes modulate gene transcription, and can be inherited in a parent-of-origin-specific manner [12, 13]. Methylation of DNA cytosine residue at the carbon 5 position is a common epigenetic feature and is often found in the sequence context CpG. Methylated DNA in gene promoters typically represses the corresponding gene [14]. Modified local and global DNA methylation patterns are found in the placenta of newborns with low birth weight [15, 16]. The DNA methylation profile of mice placenta reflects the fetal growth and development with a potential impact on the offspring's health.

We hypothesized that a GDM-inflicted environment could negatively affect placental function. We established a GDM mouse model of intrauterine hyperglycemia described by Ding et al. (2012) [2] to study how the intrauterine environment of GDM affects the placenta in both first and second filial generations.

## RESULTS

### Placental and fetal weight in F1-offspring and F2-offspring of intrauterine hyperglycemia

**Figure 1:**
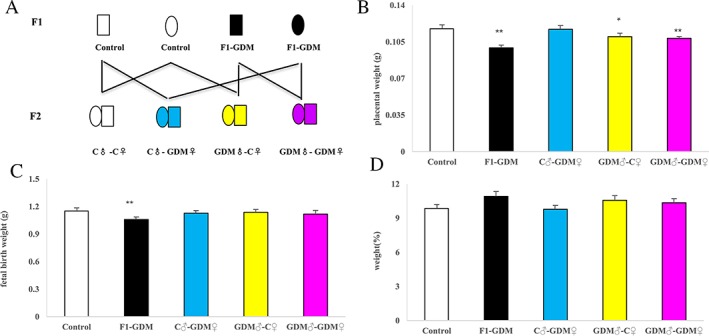
Experiment design, placental weight, fetal weight, fetal/placental weight of mice for control group (C), F1-GDM group, F2-GDM group **(A)** Experimental design. Circles designate females and squares designate males. Note that mating pairs were non-siblings. **(B)** Placental weight of all five groups. **(C)** Fetal weight of all five groups. **(D)** Ratio of fetal weight over placental weight of all five groups on. In (B)-(D), data are presented as mean ± SE (n>20 mice per group).

We established a GDM mouse model by inducing hyperglycemia after pregnancy, and female and male F1 adults of control and GDM mice were intercrossed to obtain F2 offspring (Figure 1A). Placenta weight and fetal weight in F1-GDM mice was less than control offspring (0.099 ± 0.002 g, p < 0.01; 1.058 ± 0.027 g, p < 0.01). However, in the F2-GDM offspring, placental weight decreased in only the GDM♂-C♀ group (0.110 ± 0.003 g, p < 0.05) and the GDM♂-GDM♀ (0.108 ± 0.002 g, p < 0.01) (Figure 1B). Fetal weight was not different between the three F2-offspring (Figure 1C). No matter whether the phenotype changed or not on F1-GDM offspring or the F2-GDM offspring, there were no significant difference from the aspect of weight ratio (fetal birth weight/placental weight) (Figure 1D). The results suggested that intrauterine hyperglycemia had an intergenerational effect on mouse placenta.

### Effects of intrauterine hyperglycemia on global placental expression patterns in both f1 and f2 offspring

**Figure 2:**
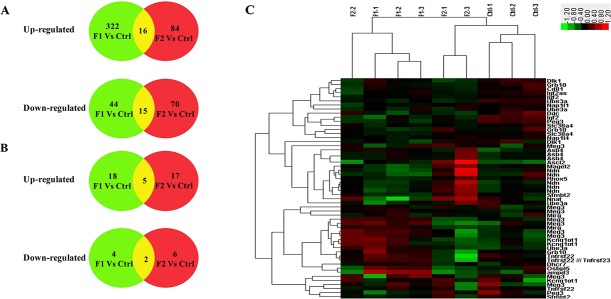
The basic statistic results of the genes identified in placenta both in 3 groups according to microarray results (control group: C1, C2, C3; F1-GDM group: F1-1, F1-2, F1-3; F2-GDM groups (GDM♂- GDM♀): F2-1, F2-2, F2-3) **(A)** the number of identified differentiated probes including imprinted and non-imprinted genes; **(B)** the number of identified differentiated imprinted genes probes; (red: up-regulated genes; green: down-regulated genes; yellow: the feature-selected genes). **(C)** Hierarchical clustering of differentially expressed imprinted genes. Clustering was based on the expression levels of genes that were analyzed by the feature selection. Bar color represents a logarithmic scale from -3.0 to +3.0.

Microarray analysis showed distinct gene expression patterns for the F1-GDM and F2-GDM groups (Figure 2A). The F1-GDM group had 366 genes (~2% of the total) whose expression was changed by >1.5 fold with P<0.05 or by <0.5 fold with P<0.05, which was comprised of 322 up-regulated genes and 44 down-regulated genes (Supplementary Table 2). The F2-GDM group had 154 genes (~1.5% of the total) whose expression was changed by >1.5-fold with P<0.05, including 84 up-regulated genes and 70 down-regulated genes placentae (Supplementary 1). Between F1-GDM and F2-GDM placenta, there are only 16 up-regulated genes and 15 down-regulated genes compared with control group. Intrauterine hyperglycemia has different effects on gene expression in the placenta of F1 and F2 offspring. Real-time quantitative PCR analysis of 15 select genes confirmed the microarray results (Supplementary Figure 1).

To investigate the influence of intrauterine hyperglycemia on imprinted genes, we selected differentiated genes with a fold change > 1.2 fold and P < 0.05 or less than 0.8 with P<0.05. This revealed 35 up-regulated genes (18 genes in the F1-GDM group and 17 genes in F2-GDM) and 10 down-regulated genes (4 genes in the F1-GDM group and 6 genes in F2-GDM) (Figure 2B, Supplementary Table 3). The heat map in Figure 2C was based on hierarchical clustering of 9 samples (3 control samples, 3 F1-GDM samples, 3 F2-GDM samples), which exhibited a direct concept of expression alteration. Five imprinted genes were altered in the same way in both groups, including Gtl2/Meg3, Asb4, Ube3a, Ascl2, Peg3 (Supplementary Table 3). After multiple probes in microarray analysis, only Gtl2/Meg3 showed the same change in F1-GDM and F2-GDM placentae.

### Bioinformation analysis

**Figure 3:**
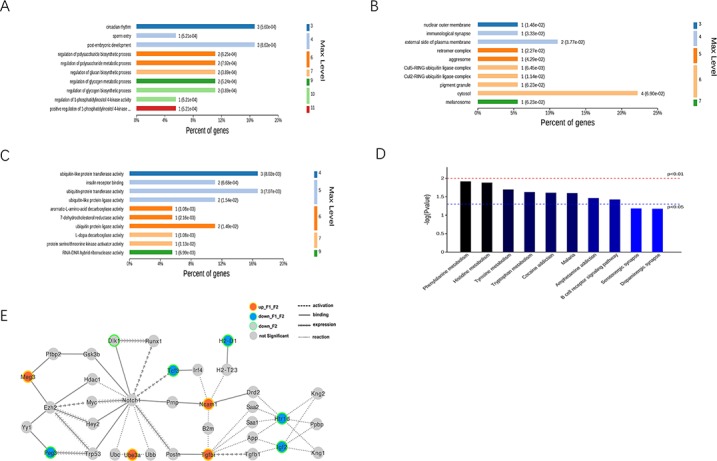
Bio-information analysis for both F1-GDM and F2-GDM groups **(A, B, C)** GO annotation of total identified differentiated expressed imprinted genes in F1-GDM or F2-GDM (GDM♂- GDM♀) in three categories; (A) biological process (BP); (B) cellular component (CC); (C) molecular function (MF); **(D)** distribution of enriched KEGG pathway. **(E)** A dysfunctional regulation gene network was constructed based on the gene-gene (or protein-protein) interactions from KEGG, String and Starbase. Columns refer to related pathways, which are colored with gradient colors from midnight blue (smaller p-value) to lighter blue (bigger p-value) in the comparison between F1-GDM group and Control group or F2-GDM group and Control group.

The differentiated imprinted gene data was analyzed using gene ontology (GO) analysis to extract information relevant to involved pathways. In the biological process (BP) analysis, the majority of genes were classified into circadian rhythm, post-embryonic development, regulation of polysaccharide metabolic process, and regulation of glycogen metabolic process (Figure 3A). The cell component (CC) analysis showed that most genes affected the cytosol (Figure 3B). Molecular functional (MF) classification revealed that most genes were involved in ubiquitin-like protein transferase activity, insulin receptor binding, and ubiquitin protein ligase activity (Figure 3C).

The KEGG pathway enrichment analysis of DEGs also provided insight into the cellular pathways associated with these DEGs. Seventeen pathways, including phenylalanine metabolism, histidine metabolism, and tyrosine metabolism, corresponded to differentially-expressed imprinted genes in F1-GDM placenta and F2-GDM placenta.

### Common modulating network in F1-GDM and F2-GDM

A dysfunctional regulation gene network was constructed based on the gene-gene (or protein-protein) interactions from KEGG, String and Starbase (Figure 3E). The imprinted gene Gtl2/Meg3 was upregulated in both F1 and F2 offspring of GDM groups. Meg3 is expressed predominantly from the maternal allele, in a reciprocal pattern from the nearby Dlk1 (delta-like 1 homolog) gene. Meg3 binds the PRC2 chromatin modification complex (including gene product of Ezh2) in mouse embryonic stem cells, and controls Dlk1 expression at the Dlk1-Meg3 imprinted locus [17]. Dlk1 expression is down-regulated in F2 GDM, supporting the dysregulation of Meg3-Dlk1 imprinted genes.

Meg3-Dlk1 imprinted genes may downregulate downstream genes, such as TGFB1, Igf2, etc, which are also differentially expressed in both F1 GDM and F2 GDM. Meg3 overexpression inhibit the TGF- β1 -stimulated cell proliferation and induced apoptosis [18], and because high glucose can stimulate TGFBI expression [19], decreasing Meg3 may stimulate TGFBI expression in an intrauterine hyperglycemic environment.

Igf2 expression was down-regulated in placenta of F1-GDM and F2-GDM groups, which is similar to former observations that the imprinted genes Igf2 and H19 were down-regulated in pancreatic islets in both F1 and F2 offspring of GDM [2]. In addition, low Igf2 levels predict weight gain in subjects with T2D [20]. The association between Meg3 and Igf2 expression was observed in both F1 and F2 generations, and DLK1/Meg3 has been associated with IGF2 [21]. These results suggest abnormal Meg3/Dlk1 expression is associated with developing diabetes.

### Dlk1 and Gtl2 expression in intrauterine hyperglycemia

**Figure 4:**
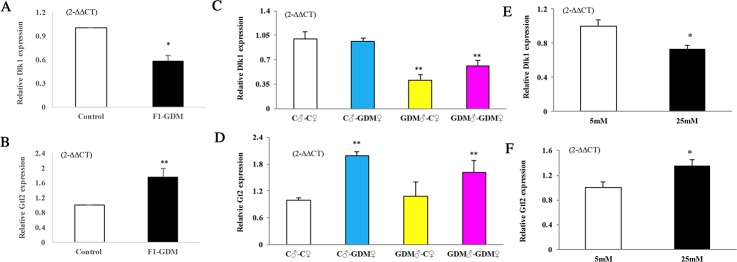
Dlk1, Gtl2 gene expression assessed by real-time quantitative PCR In **(A-B)**, Dlk1 and Gtl2 expression in placentae of F1-offspring on D18.5 (n=9 mice on for both F1-Control and F1-GDM offspring). In **(C-D)**, Dlk1 and Gtl2 expression in placentae of F2-offspring on D18.5 (n=6 mice for every group of F2 offspring). In **(E-F)**, Dlk1 and Gtl2 expression cultured in medium containing indicated concentrations of glucose of 24h (n=4 replicates/group) in at least three independent isolations. Data were analyzed with the Eq. 2-^ΔΔCT^, where ΔΔCT = ΔCT (treatment group) -ΔCT (control group), and ΔCT = ΔCT (sample) - ΔCT (internal control). The values were presented as relative expression levels of mRNA. In all panels, data are presented as mean ± SE, ^*^P<0.05, ^**^P<0.01. Significance was determined by Student t test in F1 offspring and ANOVA in F2 offspring.

According to the microarray assay, Dlk1 and Gtl2 were differentially expressed in both F1-GDM and F2-GDM placentae. Dlk1 and Gtl2 are closely associated with placental arborization and development of the spongiotrophoblastic and labyrinthine layers [22]. We found that Dlk1 mRNA levels were lower in F1-GDM placenta than in the control group (Figure 4A). However, the relative Gtl2 mRNA level was higher (Figure 4B). In the F2 offspring of intrauterine hyperglycemia, relative Dlk1 mRNA levels decreased in the GDM♂-GDM♀ and GDM♂-C ♀ groups (Figure 4C). Relative Meg3/Gtl2 mRNA levels were higher in C♂-GDM♀ and GDM♂-GDM♀ placentae (Figure 4D). These results suggest the imprinted gene had transmitted to next generation.

In order to verify the direct effect of high-glucose on placental development, we collected mouse primary trophoblast cells at 14.5 dpc and cultured in a medium containing different concentrations of glucose for 24h. High glucose (25 mmol/L) decreased Dlk1 mRNA levels in trophoblast cells, whereas Gtl2 mRNA levels increased (Figure 4E, 4F).

### Intrauterine hyperglycemia induced hypermethylation of Dlk1-DMR and hypomethylation of IG-DMR and Gtl2-DMR in placenta

**Figure 5:**
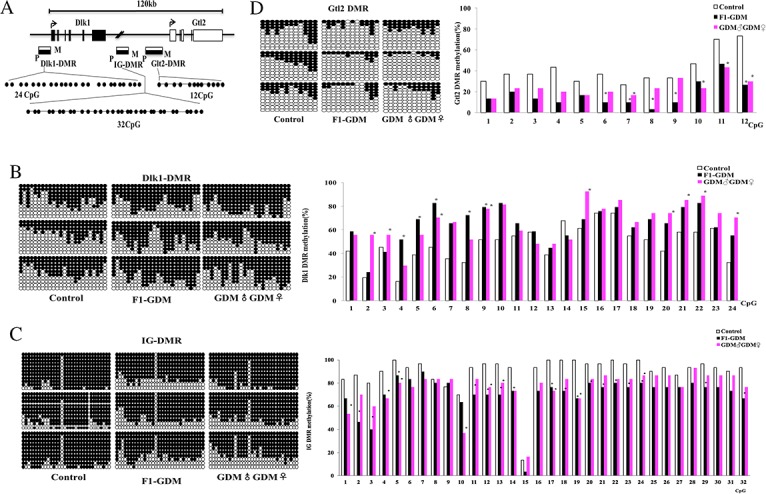
Methylation analysis of Dlk1/Gtl2 DMRs by bisulfate genomic sequencing PCR **(A)** Schematic representation of mouse imprinted locus, showing the relative position of the Dlk1 and Gtl2 genes and indicating the location of the three DMRs known to contribute to Dlk1/Gtl2 imprinting. Exons are known as black (Dlk1 gene) and white (Gtl2 gene) rectangles with arrows for transcription start sites. The locations of the three DMRs within the Dlk1/Gtl2 imprinted locus represented by boxes are shaded to indicate preferential methylation of the maternal (M) or paternal (P) allele in each region. **(B)** Methylation status of individual DNA strands of Dlk1-DMR containing 24CpG sites and the average methylation ratio in each CpG site. **(C)** Methylation status of individual DNA strands of IG-DMR containing 32CpG sites and the average methylation ratio in each CpG site. **(D)** Methylation status of individual DNA strands of Gtl2-DMR containing 12CpG sites and the average methylation ratio in each CpG site. Ten clones per mouse; a total of 30 clones per group were sequenced. Each line represents the sequence of a single clone. CpG sites were shown as blank (unmethylated) or filled black (methylated) circles. In the histograms, results were expressed as methylation percentage of each CpG site. (n=3 mice per group). ^*^p<0.05 vs control (χ^2^ test).

As imprinted genes, Dlk1 and Gtl2 allelic expression in mice is mainly regulated by allele-specific methylation at three DMRs. We collected placentae from 18.5dpc mice from the control, F1-GDM, and GDM♂-GDM♀ groups. We analyzed methylation levels of 24 CpGs of the Dlk1-DMR, 32 CpGs of the IG-DMR, and 12 CpGs of the Gtl2-DMR by bisulfate genomic sequencing PCR (BSP) (Figure 5A).

Dlk1-DMR methylation levels were higher in the F1-GDM and GDM♂-GDM♀ groups than the control group (Figure 5B). Analyzed by BSP, IG-DMR was highly methylated in control group (90% methylated), but not in the F1-GDM and GDM♂-GDM♀ groups (Figure 5C). Gtl2-DMR was less methylated in the F1-GDM and GDM♂-GDM♀ groups than in the control group (Figure 5D). These results indicate that intrauterine hyperglycemia could lead to Dlk1-DMR hypermethylation and IG-DMR and Gtl2-DMR hypomethylation in both F1 and F2 offspring.

## DISCUSSION

Hyperglycemia during pregnancy may lead to low birth weight [23, 24], and low birth weight is associated with an increased risk of obesity, diabetes, and cardiovascular diseases during adult life [25]. We found that intrauterine hyperglycemia induced lower fetal weight and placental weight in F1-offspring. Most GDM mice were susceptible to intrauterine hyperglycemia early in the pregnancy, which caused poor fetal nutrition [26]. Undernutrition reduced birth weight, impaired glucose tolerance and obesity in both first- and second- generation offspring [27]. In F2 offspring, placental weight decreased through the paternal line, but not the maternal line. Paternal phenotype transmission might be associated with the susceptibility of sperm in an intrauterine hyperglycemic environment [2].

According to the microarray analysis, intrauterine hyperglycemia affected 37 imprinted genes (Supplementary 2). H19-Igf2 and Gtl2-Dlk1 have complementary functions in the mouse placenta [28–30]. We focused on the imprinted gene Dlk1 and Gtl2. In our study, down-regulated Dlk1 expression and up-regulated Gtl2 expression was present in F1 and F2 offspring. In the F2 offspring, decreased Dlk1 levels were passed paternally, while increased Gtl2 levels were passed maternally. Dlk1 protein includes transmembrane protein and soluble protein, which promotes the activation of the insulin/IGF-I signaling pathway and adipogenesis inhibition [29, 31]. Increased Meg3 expression may suppress TGF-β and notch signaling pathway genes, such as TGFBI. That may lead to abnormal differentiation of the pancreas, which is an essential organ for insulin secretion.

Genomic imprinting regulates the expression of a large group of monoallelically expressed genes in a parent-of-origin specific manner. Allele-specific DNA methylation occurs at differentially methylated regions (DMRs) of these genes. Allelic expression of imprinted genes Dlk1 and Gtl2 is regulated by allele-specific methylation at three DMRs: Dlk1DMR, IG DMR, and Gtl2 DMR [32–34]. We examined all three Dlk1/Gtl2 DMRs in placenta of F1-GDM and GDM♂-GDM♀ mice on embryonic day 18.5. Compared with control group, methylation levels were higher at Dlk1-DMR, and lower at IG-DMR and Gtl2-DMR in F1-GDM and GDM♂-GDM♀ placentae. These results suggested that in F1 and F2 offspring after intrauterine hyperglycemia, altered Dlk1 and Gtl2 expression in placenta is associated with altered DMR methylation.

By establishing a GDM mouse model, we found that intrauterine hyperglycemia reduced the placental weight in both F1-GDM and F2-GDM offspring, and was paternally transmitted to the next generation. Intrauterine hyperglycemia alters imprinted genes, and could cause abnormal Dlk1 and Gtl2 expression related to dysregulation of Dlk1-Gtl2 methylation in placenta of both F1 and F2 offspring. This epigenetic alteration has an intergenerational effect, which could explain adult-originated disease in the GDM offspring.

## MATERIALS AND METHODS

### Animal care and model

The animal care and treatment protocols complied with institutional guidelines for laboratory animals established and approved by the Institutional Animal Care and Use Committee (IACUC), School of Medicine, Zhejiang University. Eight-week-old virgin female ICR mice (n=50) were mated with normal males. The mice were housed under a 12:12 h light/dark cycle at 25 ± 0.5°C with 50-60% humidity, and were fed ad libitum with a standard diet and water.

A copulation plug present after overnight mating determined pregnancy onset (defined as day 0 (D0) of pregnancy), and the females were randomly divided into a control group and an intrauterine hyperglycemia group with GDM (GDM group). After 12-h fast, Mice in the GDM group were induced with a single intraperitoneal injection of streptozotocin (STZ, Sigma, St. Louis, MO) in 0.1 mM citrate buffer (pH4.5) at a dose of 150 mg/kg body weight (BW). Mice in the Control group received an equal volume of citrate buffer. On D3 of pregnancy, diabetes was confirmed by measuring blood glucose concentration through the tail vein and determined as a glucose level above 16.7 mM (300 mg/dl). Maternal blood glucose was remeasured on D7 and D20 of pregnancy to confirm the diabetic condition as previous described [35, 36].

Every litter size was controlled to 10-12 at birth to assure uniformity. The GDM pups were fostered by normoglycemic females until they were three weeks old. The female (♀) and male (♂) F1 adults of control (F1-Control) and GDM (F1-GDM) mice were intercrossed to generate F2 offspring, including 4 groups: 1) C♂-C♀, 2) C♂-GDM♀, 3) GDM♂-C♀, 4) GDM♂- GDM♀.

### Placenta collection and in-vitro primary trophoblast cell cultivation

The placentae were obtained and weighed at D18.5 from the pregnant mice in the control, F1-GDM, and F2-GDM groups. We used placentas 14.5 days post coitum (dpc) to isolate primary trophoblast cells as previous described [37]. Isolated trophoblast cells were incubated in RPMI-1640 containing different glucose concentrations (5 mmol/L or 25 mmol/L) for 24h (37°C).

### RNA isolation, microarray analysis of placenta

We obtained placentae of control, F1-GDM, and GDM♂-GDM♀ groups (3 placentae per group) near term (18.5 dpc), each from a different litter. Placental RNA was isolated by TRIzol Reagent (Invitrogen Life Technologies, Carlsbad, CA, USA). After first-strand and second-strand cRNA preparation, placental cRNA was processed for use on the Affymetrix Mouse 430 2.0 array (Affymetrix, Santa Clara, CA) according to the manufacturer's instructions.

Data were analyzed with Affymetrix GeneChip Operating Software Version1.4. Comparisons were made between control group and F1-GDM group, and between control group and F2-GDM group. Each placental sample was processed and analyzed independently. After normalization with dChip (http://www.dchip.org/) estimation, the fold change and statistical significance (q-values) were calculated with SAM version 2.23. Genes with more than 1.5-fold or less than 0.5-fold change with P < 0.05 were further analyzed by Molecule Annotation System and Cluster 3.0.

### Gene expression (quantitative PCR)

Expression of select placental genes was verified by real-time quantitative PCR. Total placental RNA was isolated using RNAiso (TaKaRa, Japan), and cDNA was synthesized using oligo-dT and random primers (TaKaRa, Japan) for real-time quantitative PCR (ABI Prism 7900HT; Applied Biosystems, Foster City, CA). GAPDH was the internal control. Full list of primer sequences is shown in Supplementary Table 1.

### Bioinformatics analysis

The multi-omics data analysis tool, OmicsBean (http://www.omicsbean.com:88/), was used to assign biological functions, subcellular locations, and molecular functions to each gene based on Gene Ontology (GO) categories. The overlaps between the lists of DEGs were detected by Fisher's exact test. P-value denotes the significance of a GO term enrichment in DEGs clusters and/or pathway correlations (P-value < 0.05 was considered significant). The Kyoto Encyclopedia of Genes and Genomes (KEGG) pathway analysis was performed in order to enrich high-level functions in the defined biological systems.

### Gene interaction network construction

Gene interactions from String database [38] and KEGG database [39] were used to annotate the gene interaction network between the differentially expressed genes in F1-GDM and F2-GDM mice. For protein-protein interactions (PPIs) in String database, PPIs with confidence score over 0.7 were taken into consideration. For non-coding RNAs in differentially expressed genes, the protein-RNA interactions were predicted using StarBase v2.0 [40]. For all gene-gene interactions, Cytoscape was used to construct the gene-gene interactions.

### DNA methylation (bisulfite genomic sequencing PCR)

Genomic DNA was extracted from placenta of D18.5 mice in the control, F1-GDM, and GDM♂-GDM♀ groups. Bisulfite was converted using the EpiTect bisulfite kit (Qiagen, Valencia, CA) according to the manufacturer's instructions to deaminate cytosine to uracil; 5-methyl-cytosine was protected from deamination. The methylation status of Dlk1 differentially methylated region (DMR), Gtl2-DMR, and intergenic differentially methylated region (IG-DMR) was determined by cloning and sequencing of bisulfite-treated DNA. The full list of primer sequences is shown in Supplementary Table 2. The purified PCR products were cloned using the pMD 19-T vector system (Takara, Dalian, China). The cloned sequence was analyzed with 3730 DNA Analyzer Polymers (Applied Biosystems, Carlsbad, CA).

### Statistical analysis

Data were analyzed using SPSS 16.0, and were presented as mean ± SE. Statistical analysis including unpaired two-tailed Student's *t*-test, one-way analysis of variance (ANOVA), or Chi-square test were performed as described in the figure legends. P < 0.05 was considered statistically significant.

## SUPPLEMENTARY MATERIALS FIGURE AND TABLES






