# Supplementary material for: Intrauterine hyperglycemia induces intergenerational Dlk1-Gtl2 methylation changes in mouse placenta
Source: Oncotarget. 2018 Jan 5;9(32):22398–405. doi: 10.18632/oncotarget.23976 (PMC5976473; doi:10.18632/oncotarget.23976)
Supplement: Supplementary file 1 [file oncotarget-09-22398-s001.pdf]

Intrauterine hyperglycemia induces intergenerational Dlk1-Gtl2 methylation changes in mouse placenta

SUPPLEMENTARY MATERIALS

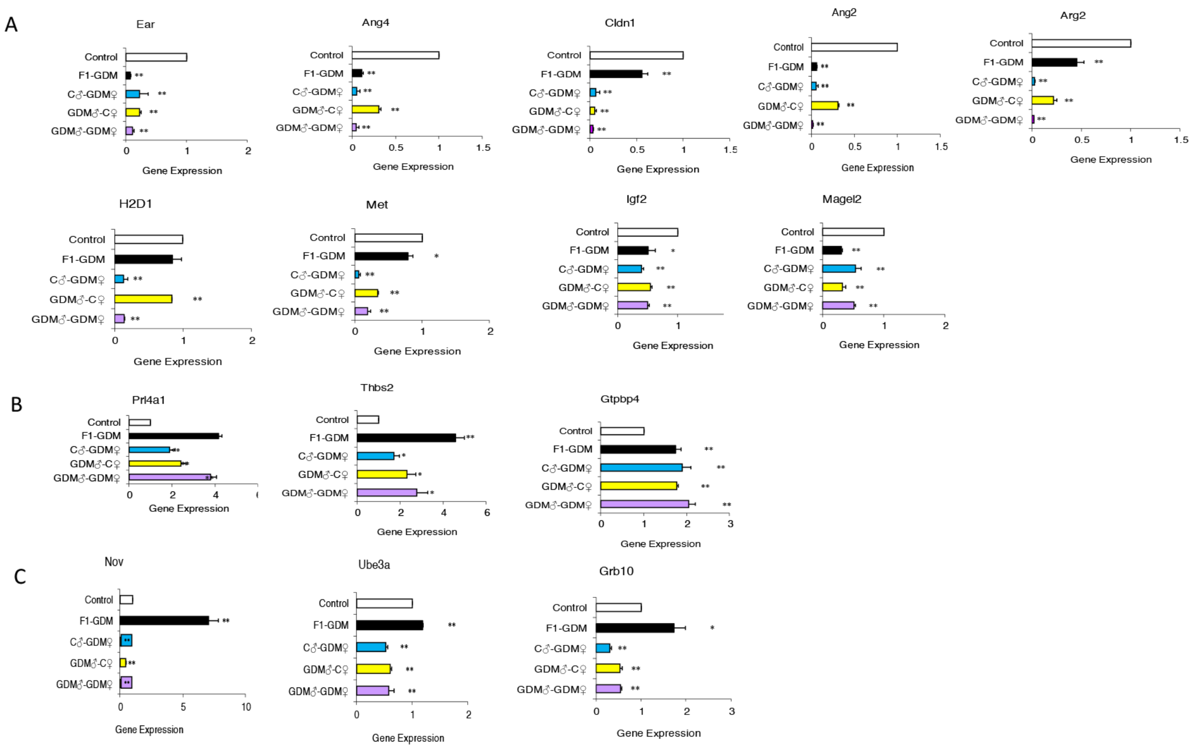

Supplementary Figure 1: Identification of fifteen differentially expressed genes by microarray analysis compared with control group. **(A)** The expression level of 9 mRNA which are down-regulated genes both in F1-GDM group and F2-GDM groups (including C♂-GDM♀ group, GDM♂-C♀ group, GDM♂- GDM♀group) (n=9 mice for control group, F1-group, F2 groups); **(B)** the expression level of 3 mRNA which are up-regulated genes both in F1-GDM group and F2-GDM groups (n=10 mice for control group, F1-group, F2 groups); **(C)** the expression levels of 3 mRNA which are diffenerentially in both F1-GDM group and F2-GDM groups compared with control group (n=9 mice for control group, F1-group, F2 groups).

Supplementary Table 1: Nucleotide sequences of primers (mouse) used for real-time quantitative PCR (SYBR Green)

Target RNA	Primers (5' to 3' direction)	Product Size	GeneBank Acc.
GAPDH-F	TGACGTGCCGCCTGGAGAAA	98 bp	NM_008084
GAPDH-R	AGTGTAGCCCAAGATGCCCTTCAG		
Dlk1-F	ACTTGCGTGGACCTGGAGAA	221bp	NM_001190703
Dlk1-R	CTGTTGGTTGCGGCTACGAT		
Gtl2-F	CCATTTGCTGTTGTGCTCAGGT	196bp	NR_003633
Gtl2-R	TGCAACGTGTTGTGCGTGAAG		
Ang2-F	TTGTTCTTGGTCTTCCTGTT	105bp	NM_007449.2
Ang2-R	TGGCTTGGCATCATAGTG		
Ang4-F	TGGAATCACTGTTGGAAGA	144bp	NM_177544.4
Ang4-R	TTGGCTTGGCATCATAGT		
Arg2-F	TCTATGACCACCTTCCTACT	111bp	NM_009705.3
Arg2-R	ACTCTGTAATGCTGTTGTGA		
Cldn1-F	CTTCAGCAGAGCAAGGTT	173bp	NM_016674.4
Cldn1-R	CATAGGCAGGACAAGAGTTA		
Ear1-F	ACCAACCTCCAATGTAATGT	174bp	NM_007894.1
Ear1-R	CCGAGATGAACTATTATGACAG		
Met-F	TGCGGTCTCAATATCAGTAG	106bp	NM_008591.2
Met-R	TGCGGTCTCAATATCAGTAG		
H2D1-F	ACGATGTGTTCAGGTCTC	177bp	NM_010380.3
H2D1-R	GCAAGGTTGGCTATGGAA		
Nov-F	GCTTCCTGCTCTTCCATC	184bp	NM_010930.4
Nov-R	GGTCTCATCTCAGAACAACT		
Emb-F	TTGCCGAAGTCATCCTCT	135bp	NM_010330.4
Emb-R	CTATGCCATTGCTATCATCTG		
Prl4a1-F	CTCCTCCTATCCTCTCCAA	134bp	NM_011165.3
Prl4a1-R	CAATTCAGACCAGGCAGTA		
Thbs2-F	GCTACTAATGCCACCTACC	140bp	NM_011581.3
Thbs2-R	TCCTTCTCATCGCTCACA		
Gtpbp4-F	AGCCTCTTATTGTTGTAGCA	149bp	NM_027000.4
Gtpbp4-R	AACTTGAATGACACCTTCCT		
Igf2-F	GCCAAGTCCGAGAGGGACGTGTCG	95bp	NM_001122736.2
Igf2-R	CAGGTGTCATATTGGAAGAACTTGC		
Magel2-F	GGTAGTGCTTGCTGAGAG	110bp	NM_013779.2
Magel2-R	GCTTGAAGGCTGAATAGGA		
Grb10-F	AAGAGGTAGGACGCAAGT	151bp	NM_001177629.1
Grb10-R	CCAGCAATCAGGTAGAAGAT		
Ube3a-F	AGAGTATGACGGTGGCTAT	208bp	NM_001033962.1
Ube3a-R	GCAAGTATGAGATGTAGGTAAC		

Supplementary Table 2: Nucleotide sequences of primes (mouse) used for bisulfite genomic sequencing PCR

DMRs	Primers (5' to 3' direction)	Product Size	CpG sites
Gtl2DMR CT-F1	TGGTTTGGGGGTAGTTTTTTATTGTAG	285bp	12
Gtl2DMR CT-R	AAAAAATACAAATAAATTAATTAACAAATCACAAA		
Gtl2DMR CT-F2	ATTTTTAAATGATGGTTGATGTGGGTTT		
Dlk1-DMR-F1	GATTAGTGATTT ATA ATTTGT GTTTTGGTT	413bp	24
Dlk1-DMR-F2	GAGATTAAGTAAGAGGTGGGA AAGGGT		
Dlk1-DMR-R	AAA CTCACCTAA ATA TACTAA AAA CAA ATA		
IG-DMR-F1	GTATGTGTATAGAGATATGTTTATATGGTA	458bp	32
IG-DMR-F2	GTGTTAAGGTATATTATGTTAGTGTTAGGA		
IG-DMR-R	GCTCCATTAACAAAATAATACAACCCTTC		

Supplementary Table 3: Common regulated differentiated imprinted genes in F1-GDM and F2-GDM placenta

Probe Set ID	Diffentiated Genes	F1 VS Ctrl	F2 VS Ctrl	Description
1433919_at	Asb4	1.36	1.41	ankyrin repeat and SOCS box-containing 4
1415931_at	Igf2	0.76	0.78	insulin-like growth factor 2
1452183_a_at	Meg3	1.50	1.23	maternally expressed 3
1426758_s_at	Meg3	1.51	1.28	maternally expressed 3
1425391_a_at	Osbpl5	1.36	0.79	oxysterol binding protein-like 5
1417356_at	Peg3	0.77	0.79	paternally expressed 3
1422039_at	Tnfrsf22	1.24	0.79	tumor necrosis factor receptor superfamily, member 22
1445727_at	Ube3a	1.53	1.24	ubiquitin protein ligase E3A
1432018_at	Ascl2	1.38	2.47	achaete-scute complex homolog 2 (Drosophila)
1422573_at	Ampd3	1.99	0.79	adenosine monophosphate deaminase 3

Supplementary 1: Differentiated genes in both F1-GDM group VS control group and F2-GDM group VS control group.

See Supplementary File 1

Supplementary 2: Differentiated imprinted genes in both F1-GDM group VS control group and F2-GDM group VS control group.

See Supplementary File 2
